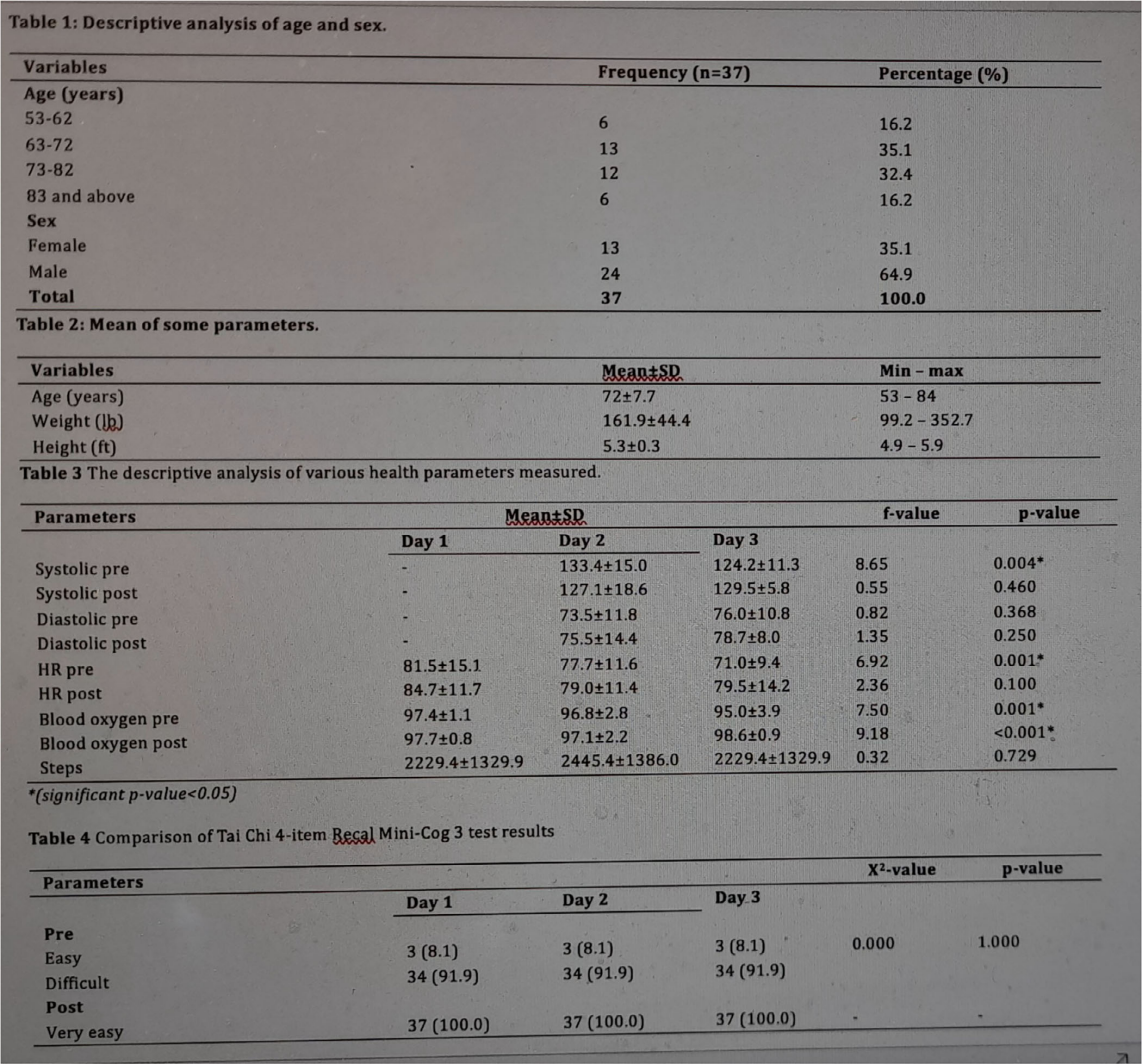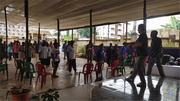# Assessing the Impact of Participatory Theatrical Exercises on Cognitive Function and Cardiometabolic Health Profile in Elderly Individuals with Dementia in Southeast Nigeria

**DOI:** 10.1002/alz70858_101026

**Published:** 2025-12-25

**Authors:** Chukwuanugo Ogbuagu, Sornchai Chatwiriyachai, Ekenechukwu Ogbuagu, Josephine Anenih, Uzoma Okereke, Irene Okeke, Chimnemelum Ogbuagu, Chijioke Ezenyeaku, Richard Uwakwe

**Affiliations:** ^1^ Nnamdi Azikiwe University Teaching Hospital (NAUTH), Nnewi, Nigeria; ^2^ Malongdu Theatre, Garden City Lagoon, Prachacheun Rd., Laksi Bangkok, Thailand; ^3^ Nnamdi Azikiwe University Teaching Hospital (NAUTH), Nnewi, Anambra, Nigeria; ^4^ Alzheimer's Disease Foundation, Ignatius Akubude Center off Amawbia Bypass, Amawbia Awka South LGA, Amawbia, Anambra, Nigeria; ^5^ University of Texas at Arlington, 701 S. Nedderman Drive Arlington, TX 76019, Arlington, TX, USA; ^6^ Nnamdi Azikiwe University Teaching Hospital, Nnewi, Anambra, Nigeria

## Abstract

**Background:**

Dementia remains a global health concern, and its prevalence is rising due to the increasing aging population. Non‐pharmacological interventions, including participatory theatrical exercises (PTE), are gaining attention for their potential to enhance cognitive function and overall well‐being in individuals with dementia. PTEs are cost‐effective and culturally adaptable, making them accessible to poor communities with limited healthcare resources.

**Method:**

We pretested the PTE on Thailand's elderly cohort before deployment to Nigeria's elderly populations in a descriptive cross‐sectional study to assess the impact of participatory theatrical exercise on cognitive function and cardiometabolic health profile of 37 selected geriatric patients with dementia in Southeast Nigeria using Tai Chi.

**Result:**

Out of 37 participants, 64.9% were male, and 35.1% were female, with an average weight of 161.9±44.4 lb and a height of 5.3 feet. Statistically significant improvements were observed in systolic blood pressure, heart rate, and blood oxygen levels over three days, with systolic BP notably decreasing from Day 2 to Day 3. Physical activity positively influenced cognitive performance, as participants rated tasks as “Very easy,” and a significant relationship was noted between steps and cognitive ease.

**Conclusion:**

The findings suggest that participatory theatrical exercises can be an effective non‐pharmacological intervention for dementia in elderly populations across different cultural contexts. These interventions promote cognitive stimulation and cardiometabolic health, foster emotional well‐being, and enhance social interactions that contribute to the overall quality of life in dementia patients.